# Autumn migration tracks of *Helopsaltes* grasshopper‐warblers from Northeast Asia support recent taxonomic assignments

**DOI:** 10.1002/ece3.9932

**Published:** 2023-03-23

**Authors:** Yuri Sleptsov, Pavel Ktitorov, Philip D. Round, Wieland Heim

**Affiliations:** ^1^ Institute of Biological Problems of the North Magadan Russia; ^2^ Department of Biology, Faculty of Science Mahidol University Bangkok Thailand; ^3^ Institute of Landscape Ecology University of Münster Münster Germany; ^4^ Department of Biology University of Turku Turku Finland; ^5^ Present address: Swiss Ornithological Institute Sempach Switzerland

**Keywords:** East Asian flyway, geolocation, *Helopsaltes certhiola*, *Helopsaltes ochotensis*, *Locustella*, *subcerthiola*

## Abstract

Migration strategies are genetically inherited in most songbirds, and closely related species can exhibit markedly contrasting migration programs. Here, we investigate the autumn migration of one *Helopsaltes* grasshopper‐warbler from a population near Magadan, North East Russia, based on light‐level geolocation. Although often considered to belong to Middendorff's Grasshopper‐warbler *H. ochotensis*, recent genetic studies suggest that birds from this population are more closely related to Pallas's Grasshopper‐warbler *H. certhiola*. We compare the migratory behavior of the Magadan bird with two Pallas's Grasshopper‐warblers tracked from populations in the Kolyma River valley and the Amur region, Russia. We found similar migration patterns in all three tracked individuals, with stopover sites in eastern China and wintering sites in mainland Southeast Asia, within the known range for Pallas's Grasshopper‐warbler. Furthermore, based on morphological data compiled during bird ringing, we were able to confirm the presence of potential “Magadan grasshopper‐warblers” during spring and autumn migration in Thailand. Our scant data provide further evidence that Magadan *Helopsaltes*, notwithstanding their morphological resemblance to Middendorff's Grasshopper‐warbler, constitute a population of Pallas's Grasshopper‐warbler.

## Graphical abstract

Here, we investigate the autumn migration of Helopsaltes grasshopper‐warblers from North‐East Russia based on light‐level geolocation. We found that individuals from the Magadan population showed similar migration patterns to Pallas's Grasshopper‐warblers, with stopover sites in eastern China and stationary sites during the boreal winter in Southeast Asia. Although limited to single individuals, our data provide further evidence that grasshopper‐warblers from Magadan should be included into Pallas's Grasshopper‐warbler and should not be treated as a subspecies of Middendorff's Grasshopper‐warbler.

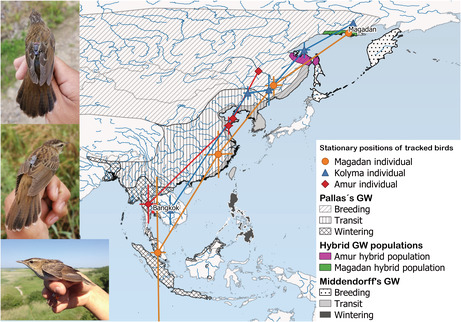

## 1 INTRODUCTION

Until recently, the migration strategies of small landbird species of the East Asian flyway were relatively poorly studied in comparison with those of the European‐African and the American flyways (McKinnon & Love, 2018). This changed considerably during the last decade following the development of tracking techniques based on light levels (see Yong et al., 2021 for review). Tracking studies of East Asian landbirds showed that while a few species of small‐ and medium‐sized species undertake direct sea crossings (Koike et al., 2016) most generally avoid prolonged marine transits, performing detours around water barriers even when they start migration from the islands of the Pacific Ocean (Ktitorov et al., 2022; Yamaguchi et al., 2021; Yamaura et al., 2017). Two main migration corridors of East Asian landbirds were found: first, a mainland route through continental East Asia for populations spending the nonbreeding season in India or mainland Southeast Asia, and second, an island route, with birds migrating from North‐East Asia through the chain of islands in the Pacific to nonbreeding sites on the Philippines or Indonesia (Heim et al., 2020). A few species deviate from these general patterns. For example, a Blue‐and‐white Flycatcher *Cyanoptila cyanomelana* was tracked from its breeding grounds in mainland Russian Far East to a wintering site on the islands of the Philippines (Heim et al., 2022), and Arctic Warblers *Phylloscopus borealis* breeding in Alaska and wintering in Philippines and Palau might follow a loop migration pattern, taking a mainland route during autumn migration, but the island route during their spring migration (Adams et al., 2022).

**FIGURE 1:**
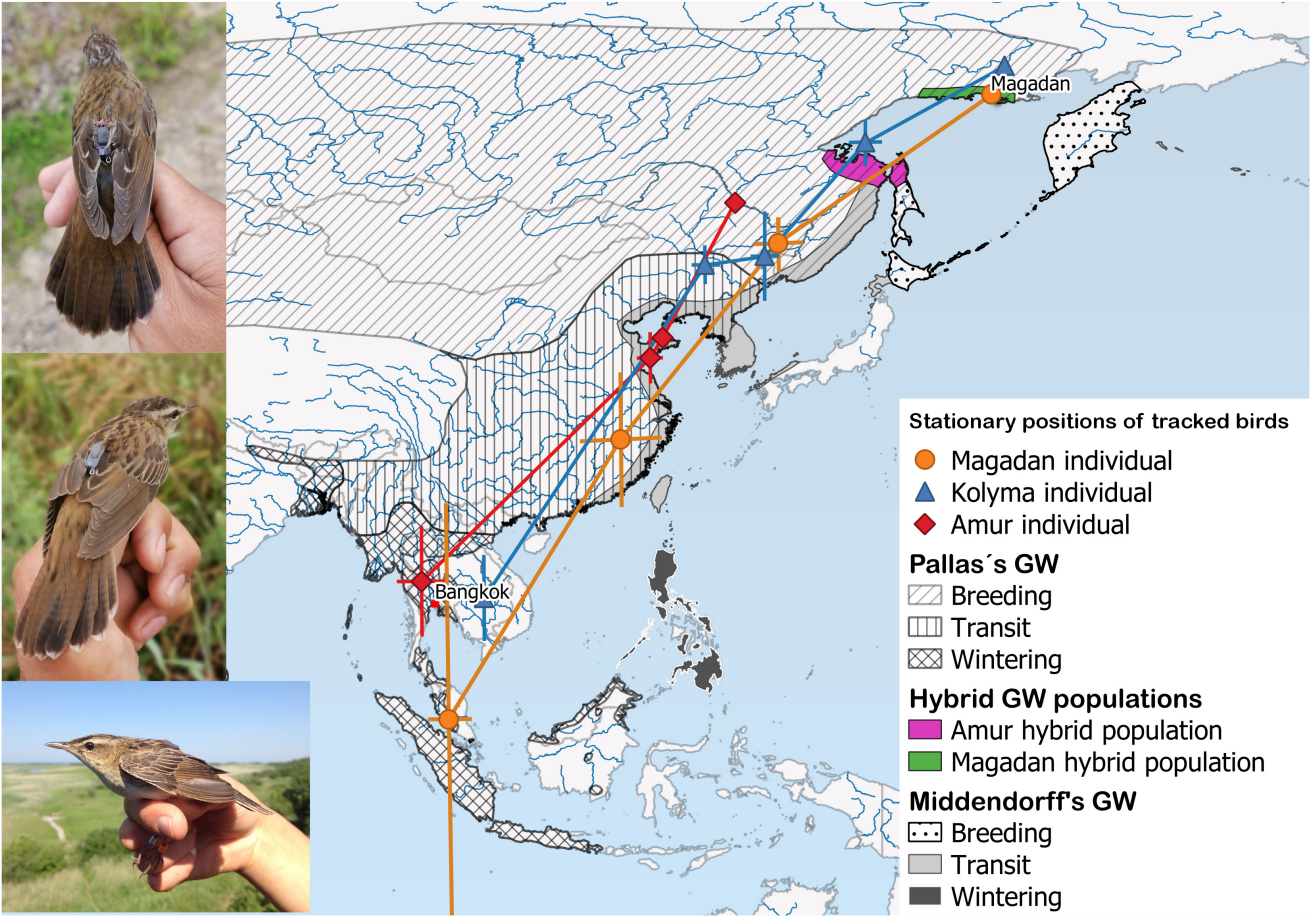
Stationary positions (plus standard deviation) during autumn migration and the boreal winter of tracked grasshopper‐warblers, based on light‐level geolocation data, in relation to the distribution ranges of Pallas's and Middendorff's Grasshopper‐warblers (data from IUCN, 2023) and hybrid populations. The inset shows (from top to bottom) a “Magadan grasshopper‐warbler,” a Pallas's Grasshopper‐warbler from the Kolyma River and a Pallas's Grasshopper‐warbler from the upper Amur River valley, fitted with geolocators.

Two closely related species that are believed to differ in their migration routes and nonbreeding locations are found within the *Helopsaltes* (*Locustella*) grasshopper‐warblers: Pallas's Grasshopper‐warbler *Helopsaltes certhiola* has a wide breeding distribution across northern Eurasia eastward to the Sea of Okhotsk and spends the boreal winter in mainland Southeast Asia and western Indonesia (Figure 1, BirdLife International, 2022; Heim et al., 2020). Middendorff's Grasshopper‐warbler *Helopsaltes ochotensis* has a limited breeding distribution around the Sea of Okhotsk and is known to spend the boreal winter on the Philippines, Borneo, and Sulawesi (Figure 1, BirdLife International, 2022; Kennerley & Pearson, 2010). The population of *Helopsaltes* from the area of Magadan, on the northern coast of the Sea of Okhotsk in North‐East Russia, (Figure 1), has sometimes, on the basis of its morphology (large size, much reduced streaking, and somber coloration) been placed as a subspecies, *H. o. subcerthiola*, of Middendorff's Grasshopper‐warbler, a taxon which otherwise breeds in the Kamchatka peninsula (BirdLife International, 2022; Gill et al., 2022; Kennerley & Pearson, 2010). However, the individuals of the Magadan population display traits that accord precisely with neither Middendorff's nor Pallas's Grasshopper‐warblers. Due to their intermediacy in both plumage characters and body size, they have long been considered a possible hybrid lineage (Kalyakin et al., 1993; Sleptsov et al., 2021). Birds from Magadan are larger, less rufescent, and much less streaked than individuals of the geographically closest subspecies of Pallas's Grasshopper‐warbler *H. c. rubescens*, which occurs further to the northwest in the valley of the Kolyma River, but with which they may come into contact. However, the range of the Magadan birds is disjunct from that of topotypical *H. o. subcerthiola* from Kamchatka which they more closely resemble (Kennerley & Pearson, 2010). Genetic studies have placed individuals from the Magadan population in the *H. certhiola* clade (Alström et al., 2018; Drovetski et al., 2015), contradicting their inclusion within *H. ochotensis* as presently implied by current major world bird checklists (BirdLife International, 2022; Gill et al., 2022). A subsequent genetic and biometrical evaluation has additionally confirmed that other contentious regional *Helopsaltes* populations, from the lower Amur River, and from northern Sakhalin, are hybrids between Pallas's and Middendorff's Grasshopper‐warblers (Evtuch, 2020).

In this study, we investigate the migration strategy of the “Magadan grasshopper‐warblers” and compare their nonbreeding spatiotemporal distribution to what is known of the winter distribution of Pallas's and Middendorff's Grasshopper‐warblers. The spatiotemporal migration program is heritable and genetically controlled, so hybrids descending from parents with different migratory orientations might be expected to show “intermediate” or “hybrid” migration patterns (Delmore & Irwin, 2014; Sokolovskis et al., 2023). In general, differences in migratory directions are important to separate otherwise closely related taxa, as different routes can, for example, imply differences in arrival at the breeding grounds that can support speciation processes (Irwin & Irwin, 2005). This allows us to make the following assumptions for the case of the North‐East Asian grasshopper‐warblers: If the birds from the Magadan population should take a continental route to wintering sites in mainland Southeast Asia or western Indonesia, this would therefore provide evidence that their genetic migration program is closely related to that of the Pallas's Grasshopper‐warbler. A route through the islands of the Pacific toward a wintering ground in the Philippines, on the contrary, would suggest that migration genes of the Magadan population were acquired as a result of gene drift from Middendorff's Grasshopper‐warblers.

## 2 MATERIALS AND METHODS

### 2.1 Geolocator tracking

We used light‐level geolocators to track the migration of grasshopper‐warblers (Intigeo W50B11, Migrate Technologies, UK) weighing 0.5 g (<4% of the birds' body weight). In 2020, we tagged nine male grasshopper‐warblers of the hybrid population near Magadan (150.89058 E, 59.56142 N) and nine males of *H. сerthiola rubescens* in the Kolyma River Valley (152.40303 E, 62.88400 N; see Figure 1 for the location of the populations). In 2021, we retrieved one geolocator from the Magadan population (BW944), which recorded data up to September 30, 2020 and one geolocator from the upper Kolyma River (BW955) containing data up to November 21, 2020. Thus, the return rate of birds with loggers was 11%. In addition, we included already published tracking data of one Pallas's Grasshopper‐warbler *H. c. certhiola* from a breeding population further southwest in the Amur region, Russian Far East (BG101) (Heim et al., 2020).

We analyzed the light data following Lisovski et al. (2020) using R version 3.4.1 (R Core Team, 2021). Twilights were defined using a threshold of 0.8 with the BAStag package (Wotherspoon et al., 2013). We adjusted the start times of the geolocators' internal clocks by adding 12 h. We used a breeding site calibration (24 June to 9 July and 5 July to 8 August in BW944 and BW955, respectively) to estimate the sun elevation angle for each device during the stationary period (Lisovski et al., 2020). Periods of residency were distinguished using the *changeLight* function with a quantile of 0.9 and 1 day as the minimum duration of stay in the GeoLight package (Lisovski & Hahn, 2012). Zenith angles (−4.05 and −5.34 for BW944 and BW955, respectively) for these periods were estimated using the function *getElevation*, and coordinates were calculated using the *coord* function. Final sites were estimated with the *mergeSites* function and a distance threshold of 200 km.

We defined the day of departure as the first day after a period of residency, and the day of arrival as the first day of stationarity of any given period. We defined stopover time as the sum of days of periods of residency during migration, whereas travel time was defined as the number of days during migration excluding periods of residency. The total duration of migration was calculated as the number of days between departure from the breeding site and arrival at the wintering site for autumn and vice versa for spring migration. Migration distance was calculated as the sum of the great‐circle distances between mean positions (including all sites of residency), whereas the direct distance was calculated as the great‐circle distance between breeding and nonbreeding site. We estimated travel speed by dividing the migration distance by the number of days spent migrating (total duration of migration minus stopover time), whereas total speed of migration was estimated by dividing the migration distance by the total duration of migration.

### 2.2 Ringing data

Given our minimal sample of tracked birds, we examined mensural and plumage data from *Helopsaltes* handled during mist‐netting and ringing operations at stopover sites around the shores of the Gulf of Thailand. Here, Pallas's Grasshopper‐warbler is a regular migrant, while Middendorff's Grasshopper‐warbler remains unrecorded (Treesucon & Limparungpatthanakij, 2018). Presumed Magadan birds were identified on the basis of their dorsal patterning (obscure streaking) and by wing‐length (measured as maximum chord) and were photographed (P. D. Round, unpublished data held on file).

Grasshopper‐warblers of the Magadan population are distinguishable from Middendorff's Grasshopper‐warblers by the presence of obscure dark streaking on their crown and mantle feathers. The presence of obscure dark streaks on the long feathers of the upper‐tail coverts in Magadan birds additionally separates them from Middendorff's, in which the upper‐tail coverts are uniform. Distinguishing some Magadan birds from Pallas's is less straightforward, but dorsal streaking is usually much more obscure in the former; they always lack any rump streaking and they are substantially larger. Birds with wing chord longer than 70 mm showing hybrid features in coloration are identifiable as male “Magadan grasshopper‐warblers,” since the other known hybrid population (from Amur and N. Sakhalin) is smaller in size with a winglength <70 mm (Evtuch, 2020; Sleptsov, 2018, Appendix 1).

## 3 RESULTS

### 3.1 Geolocator tracking

All three tracked grasshopper‐warblers were found to migrate southwestward along the mainland corridor, with stopover sites in continental East Asia and nonbreeding sites in Southeast Asia. The initial part of the migration path of birds from the north of the range ran along the northwestern coast of the Sea of Okhotsk (Figure 1). Individuals belonging to *H. c. rubescens* and *H. c. certhiola* (BW 955 and BG 101) were found to migrate to nonbreeding sites in eastern and western Thailand, respectively, while the bird from the vicinity of Magadan (BW 944) continued to Peninsular Malaysia. The two more northerly breeders, from the vicinity of Magadan and the Kolyma Valley, left their breeding sites in mid‐August, while the breeding bird from the Amur region began its migration later, in early September (Table 1). The migration distance was shortest for the Pallas's Grasshopper‐warbler from the Amur region, and longest for the Magadan bird (Table 1). Both Pallas's Grasshopper‐warblers migrated more slowly than the bird from the Magadan population (221 and 262 vs. 314 km/day). All individuals arrived at their final nonbreeding sites in September, with the bird from the Amur population having the shortest duration migration. Nonetheless, the Magadan individual, in spite of undergoing the longest migration, reached its wintering grounds much earlier than other two birds (Table 1).

**TABLE 1:** Autumn migration details of a grasshopper‐warbler from the Magadan population (BW944) compared with two Pallas's Grasshopper‐warblers from the Kolyma River valley (BW955) and the Amur region, Russian Far East (BG101), based on light‐level geolocation data. Day of departure—first day after a period of residency, day of arrival—first day of period of residency, stopover time—sum of days of periods of residency during migration, travel time—number of days during migration excluding periods of residency, total duration of migration—number of days between departure at the breeding and arrival and the wintering site for autumn and vice versa for spring migration, migration distance—sum of the great‐circle distances between mean positions (including all sites of residency), direct distance—great‐circle distance between breeding and wintering site, travel speed—total duration of migration minus stopover time, total speed of migration—dividing the migration distance by the total duration of migration, latest date—last date with geolocation data. For more details, please refer to Material and Methods.

Individual	BW944 (Magadan)	BW955 (Kolyma)	BG101 (Amur)
Departure breeding	12 August	18 August	2 September
Arrival first stopover	13 August	19 August	3 September
Departure last stopover	2 September	16 September	13 September
Travel time (days)	5	5	8
Stopover time (days)	19	25	10
Total duration (days)	24	30	18
Migration distance (km)	7533	6635	4707
Travel speed (km/day)	1507	1327	588
Migration speed (km/day)	314	221	262
Latest date	30 September	21 November	31 May

### 3.2 Ringing data from birds trapped on passage in Thailand

**FIGURE 2:**
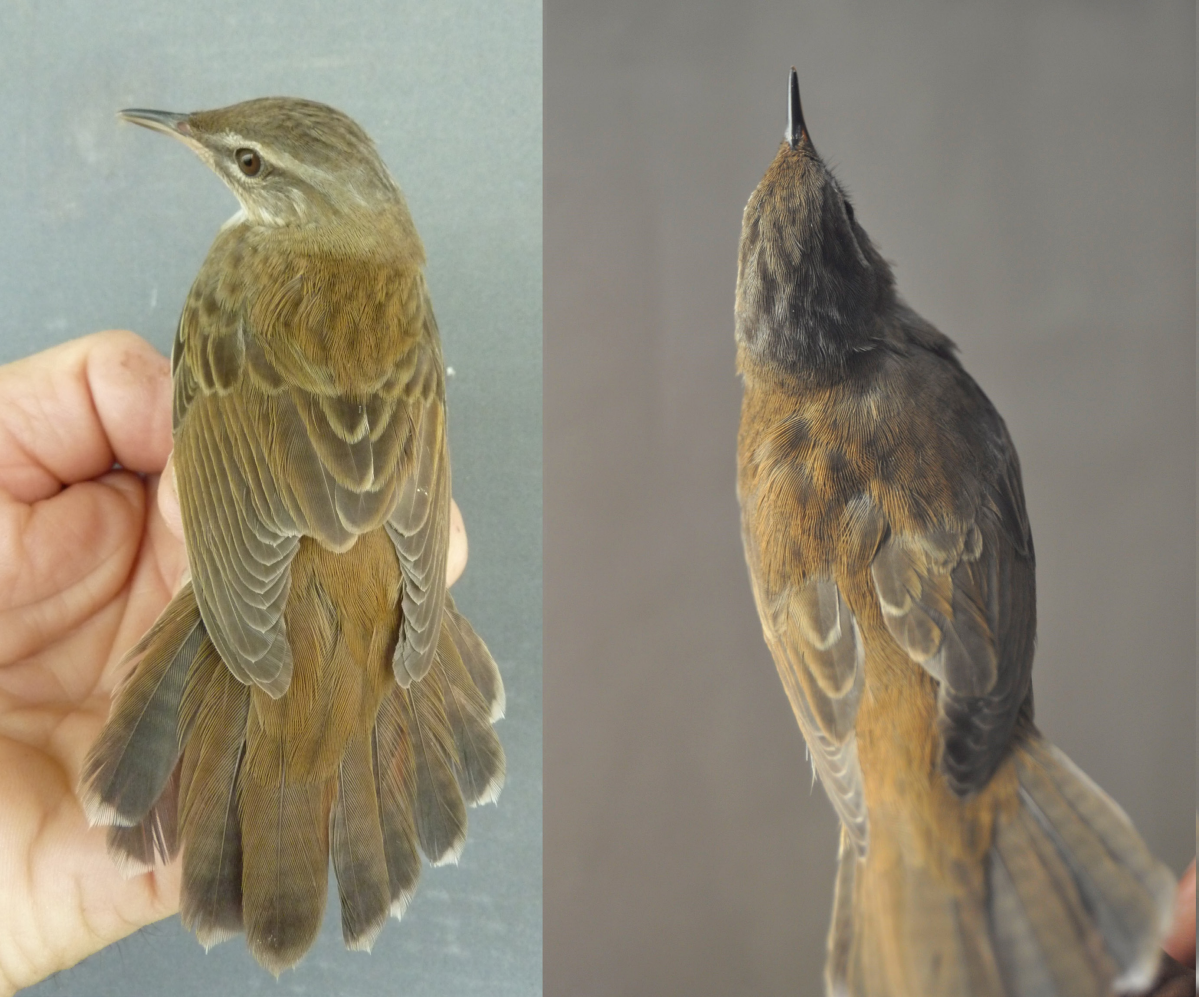
Presumed “Magadan grasshopper‐warbler” trapped on northward passage in Thailand on May 15, 2011 (left), and a grasshopper‐warbler from the breeding area in Magadan, Russia on June 18, 2014 (right). Both individuals exhibit similar identification features: obscure dorsal streaking, including on the upper‐tail coverts, and unstreaked rump. Photographs by Philip D. Round and Yury Sleptsov.

A retrospective analysis of ringing data collected from *Helopsaltes* warblers around the Gulf of Thailand, in the vicinity of Bangkok, confirmed that no individual with an undisputed phenotype of Middendorff's Grasshopper‐warbler was recorded among 303 *Helopsaltes* examined in the hand during both spring and autumn migration seasons and in midwinter. However, 15 individual *Helopsaltes* exhibiting features similar to the Magadan birds were trapped and documented by photographs (Figure 2).

From winglengths (70–72.5 mm, mean = 71 cm), nine of 15 individuals with hybrid features were identified as males, almost certainly from the Magadan population. The nine males (eight adults, one first‐winter) were mist‐netted over the years 2001–2008 during 1st to 14th of September (median date 9th of September). Two further birds with plumage features that apparently conformed to those of the Magadan population were caught on northward passage during May 14, 2011, to May 15, 2011. All emanated from a single site, the royally initiated Laem Phak Bia Environmental Research and Development Project, Phetchaburi Province, Thailand (N 13.049254, E 100.089110). While Pallas's Grasshopper‐warblers overwintered, and were widely sampled and ringed at this and neighboring sites around the Thai Gulf during the months October–April, no others that conformed to the appearance of the Magadan birds were noted.

## 4 DISCUSSION

We compiled autumn tracking data of three grasshopper‐warblers, including one “Magadan grasshopper‐warbler.” The grasshopper‐warbler from the Magadan population showed very similar migratory behavior to the Pallas's Grasshopper‐warblers from the Kolyma River valley and the Amur region (Figure 1). The migration of all observed birds followed the mainland migration corridor, as do many other East Asian landbirds (Heim et al., 2020; Yong et al., 2021). All individuals made stopovers in eastern China and migrated to wintering areas in Southeast Asia (Figure 1).

The Pallas's Grasshopper‐warbler from the Kolyma River valley most likely made its first stopover on the Schantar islands in the Sea of Okhotsk (Figure 1), suggesting that grasshopper‐warblers breeding north of that sea might cross it instead of detouring around it. This differs from Arctic Warblers tagged in Alaska, which performed a long westward detour through Chukotka and Yakutia before turning south and flying over mainland East Asia toward their wintering grounds (Adams et al., 2022).

It is noteworthy that the Kolyma and Amur Pallas's Grasshopper‐warblers overwintered in relative proximity to each other in continental Thailand, whereas the Magadan bird wintered further south in Malaysia, despite the proximity of its breeding areas to the bird from the Kolyma river valley. However, all three nonbreeding locations are within the expected range of Pallas's Grasshopper‐warbler, and hundreds of kilometers west of the known wintering range of Middendorffs' Grasshopper‐warbler (BirdLife International, 2022). The low precision of the location estimates likely stems from the grasshopper‐warblers' skulking behavior, which causes substantial shading of the geolocators' light sensor (Lisovski et al., 2012).

Based on coloration and wing measurements, we found that grasshopper‐warblers similar to the Magadan breeding birds were caught during spring and autumn migration in Thailand. The departure of the two tracked grasshopper‐warblers from the northern populations during August fits well to the observed dates of autumn migration of Pallas's Grasshopper‐warblers from the northern part of their breeding range (Andreev et al., 2006). The date of arrival of the Magadan bird (BW944) in its Malaysian winter quarters (5 September) corresponds well with the 1–14 September occurrence of the long‐winged, obscurely streaked presumed male “Magadan grasshopper‐warblers” caught on passage a little further north in Thailand. The similarity of hybrid warblers regularly ringed at Laem Phak Bia with birds breeding near Magadan, and their number, concentration and coincidence in a short period of occurrence, together suggest that our tracked Magadan bird serves as an example of a general migration pattern that is common to the grasshopper‐warblers of the Magadan population. It was expected that the bird from the Amur region might start its autumn migration later, given that its breeding site is situated further south and closer to the nonbreeding grounds. Furthermore, southern breeding populations of Pallas's Grasshopper‐warblers are known to undergo postbreeding molt on the breeding sites to a larger extent than their northern conspecifics (Eilts et al., 2021), which could also delay their migration.

The somewhat higher migration speed of the Magadan bird (Table 1) might be enabled by the generally longer wings in this population compared with most other Pallas's Grasshopper‐warblers (Evtuch, 2020; Hahn et al., 2016; Kennerley & Pearson, 2010; Sleptsov et al., 2021). However, given the very low sample size, further studies are needed to confirm this.

The overall similarity of the migration routes and the location of the nonbreeding sites of our three tracked *Helopsaltes* suggests that birds from the Magadan population likely inherited their migration program from Pallas's Grasshopper‐warbler. Our tracking data firmly support the placement of the grasshopper‐warblers of the Magadan population within the Pallas's Grasshopper‐warbler clade, in accordance with the findings of Alström et al. (2018) and Drovetski et al. (2015). We therefore call for a revision of the taxonomic status of the “Magadan grasshopper‐warbler” population and suggest its inclusion in the Pallas's Grasshopper‐warbler, contrary to the placement of this taxon in widely used world bird lists (BirdLife International, 2022; Gill et al., 2022).

## AUTHOR CONTRIBUTIONS

**Yuri Sleptsov:** Conceptualization (lead); funding acquisition (lead); investigation (lead); writing – original draft (equal); writing – review and editing (equal). **Pavel Ktitorov:** Conceptualization (supporting); visualization (equal); writing – review and editing (lead). **Philip D Round:** Investigation (supporting); writing – review and editing (equal). **Wieland Heim:** Conceptualization (supporting); formal analysis (lead); visualization (equal); writing – original draft (equal); writing – review and editing (supporting).

## Data Availability

Geolocation data used in this study are publicly available at MoveBank (www.movebank.org) in the study “Grasshopper warbler” (MoveBank ID 2667971916).

## APPENDIX 1 Wing lengths of male *Helopsaltes* grasshopper‐warblers. Given are the taxon involved, the origin of the data (published/unpublished), the sample size (*N*) as well as the mean and the range (both in mm)

**Table:**

Taxon & origin of data		*N*	Mean	Range (in mm)
Hybrid Magadan population (*H. certhiola* × *H. ochotensis*)	Sleptsov Y. 2014	34	68.5	63–72
	Sleptsov Y. 2022	13	70.23	69–72
	Evtukh G. 2020	8	72.19	69.5–74.5
Hybrid Amur population (*H. certhiola* × *H. ochotensis*)	Evtukh G. 2020	20	67.69	62–69.5
Helopsaltes certhiola rubescens	Evtukh G. 2020	12	66.98	65–69.2
	Sleptsov Y. 2018	20	65.7	62–68
Helopsaltes certhiola centralasiae	Evtukh G. 2020	9	72.62	70.3–75.2
Helopsaltes certhiola sparsimstriata	Evtukh G. 2020	35	69.8	67–73
Helopsaltes certhiola certhiola	Evtukh G. 2020	40	64.6	59–67
Helopsaltes certhiola minor	Evtukh G. 2020	26	67.8	63.4–70.9
Helopsaltes ochotensis subcerthiola	Evtukh G. 2020	42	74.9	70.7–80.6
